# Corrigendum

**DOI:** 10.2217/hep-2017-0017c1

**Published:** 2018-12-21

The Case Report by authors Roberto Iezzi, Maurizio Pompili, Eleonora Brigida Annicchiarico, Matteo Garcovich, Massimo Siciliano, Antonio Gadbarrini & Riccardo Manfredi ‘‘Hug sign’: a new radiological sign of intraprocedural success after combined treatment for hepatocellular carcinoma’, which appeared in the July 2017 issue of *Hepatic Oncology* (4)3, 69–73 (2017), was published with the authors’ names as Iezzi Roberto, Pompili Maurizio, Annicchiarico Eleonora Brigida, Garcovich Matteo, Siciliano Massimo, Gasbarrini Antonio & Manfredi Riccardo. This has now been corrected.

The authors and editors of *Hepatic Oncology* would like to sincerely apologize for any inconvenience or confusion this may have caused our readers.

